# Supplementary material for: Spherical Deconvolution of Multichannel Diffusion MRI Data with Non-Gaussian Noise Models and Spatial Regularization
Source: PLoS One. 2015 Oct 15;10(10):e0138910. doi: 10.1371/journal.pone.0138910 (PMC4607500; doi:10.1371/journal.pone.0138910)
Supplement: S1 File — (DOCX) [file pone.0138910.s001.docx]

**Appendices**

**A) Relationship with the RL-SD method**

In the limit of very high signal to noise ratio (i.e., $\mathbf{S}\circ\mathbf{Hf}^{k}/\sigma^{2}\gg n$) the modified Bessel functions ratio in Eq. (8) tends to the unity (see Fig M in **S2 File**). In that limit, Eq. (8) becomes:

$\mathbf{f}^{k+1}=\mathbf{f}^{k}\circ\frac{\mathbf{H}^{T}\mathbf{S}}{\mathbf{H}^{T}\mathbf{Hf}^{k}}$, (A-1)

which is just the undamped RL-SD method originally proposed in [[42](#_ENREF_42)] under the assumption of zero-mean Gaussian noise .

**B) Noncentral Chi noise model for SoS data - effective or standard values?**

As previously discussed, for scanners with a high number of coils where the effect of noise correlation cannot be easily decoupled, a best approximation for the noise model in SoS-based images is obtained by using effective $n_{eff}$ and $\sigma_{eff}^{2}$ values [[63](#_ENREF_63)]. This section is devoted to provide an initial insight on the implication of using a noncentral Chi noise model in our estimation with standard parameters, instead of the effective ones.

If considering the limit $n\gg1$, the ratio provided by Eq. (C-1) in Appendix C can be approximated as:

$\frac{I_{n}\left(x\right)}{I_{n-1}\left(x\right)}\approx\frac{x}{n+\sqrt{x^{2}+n^{2}}},$ (B-1)

where we have used the identity relating the expansion of a square root in terms of continued fraction. This expression can be regarded as a lower bound for the true ratio, which is more accurate insofar as $n$ increases.

Notably, based on this result we obtain

$\frac{I_{n}\left(\mathbf{S}\circ\mathbf{Hf}/\sigma^{2}\right)}{I_{n-1}\left(\mathbf{S}\circ\mathbf{Hf}/\sigma^{2}\right)}\approx\frac{\mathbf{S}\circ\mathbf{Hf}}{n\sigma^{2}+\sqrt{\left(\mathbf{S}\circ\mathbf{Hf}\right)^{2}+\left(n\sigma^{2}\right)^{2}}}$. (B-2)

Similarly,

$\frac{I_{neff}\left(\mathbf{S}\circ\mathbf{Hf}/\sigma_{eff}^{2}\right)}{I_{neff-1}\left(\mathbf{S}\circ\mathbf{Hf}/\sigma_{eff}^{2}\right)}\approx\frac{\mathbf{S}\circ\mathbf{Hf}}{n_{eff}\sigma_{eff}^{2}+\sqrt{\left(\mathbf{S}\circ\mathbf{Hf}\right)^{2}+\left(n_{eff}\sigma_{eff}^{2}\right)^{2}}}$. (B-3)

The relevant feature of these relationships is that they do not depend on the individual parameters of interest but just on their products $n\sigma^{2}$ and $n_{eff}\sigma_{eff}^{2}$. This implies that although in general the functions $I_{n}\left(\mathbf{S}\circ\mathbf{Hf}/\sigma^{2}\right)$ and $I_{neff}\left(\mathbf{S}\circ\mathbf{Hf}/\sigma_{neff}^{2}\right)$ are different, if $n_{eff}\sigma_{eff}^{2}=n\sigma^{2}$ then their ratios (which are the terms used in the computation) satisfy:

$\frac{I_{n}\left(\mathbf{S}\circ\mathbf{Hf}/\sigma^{2}\right)}{I_{n-1}\left(\mathbf{S}\circ\mathbf{Hf}/\sigma^{2}\right)}\approx\frac{I_{neff}\left(\mathbf{S}\circ\mathbf{Hf}/\sigma_{eff}^{2}\right)}{I_{neff-1}\left(\mathbf{S}\circ\mathbf{Hf}/\sigma_{eff}^{2}\right)}$. (B-4)

The accuracy of this approximation is determined by the accuracy of Eq. (B-1) and the assumption $n_{eff}\sigma_{eff}^{2}=n\sigma^{2}$. Interestingly, in [[54](#_ENREF_54)] was reported that for a system with 32 receiver channels of non-accelerated SoS-based data, the mean effective number of channels was $n_{eff}$=12. Theoretical calculations in [[63](#_ENREF_63)] predict similar $n_{eff}$ values for 32 and 16 coil systems with correlation coefficients between coil of $\rho\approx0.3$ and $\rho\approx0.2$, respectively. In a complementary analysis we have verified that Eq. (B-1) provides a ‘reasonable’ approximation for that effective number of channels. Moreover, in [[73](#_ENREF_73)] was showed that for SoS reconstructions without using fast pMRI techniques the product $n_{eff}\sigma_{eff}^{2}$ is constant across the image and equal to $n\sigma^{2}$. These results, taken together, indicate that for *non-accelerated* SoS-based data acquired in multichannel scanners with a high number of coils and moderate correlation coefficients between coils the SD estimation process may be approximately performed using the standard parameters. That is, by working in terms of the real parameters $n$ and $\sigma^{2}$, we can avoid the complex estimation of the spatial-dependent effective parameters.

**C) A note on the evaluation of the term** $I_{n}\left(x\right)/I_{n-1}\left(x\right)$

The proposed SD algorithm involves the evaluation of the ratio of modified Bessel functions of first kind. Such evaluation is best computed by considering the ratio as a new composite function, and not by means of the simple evaluation of the ratio of the individual functions. The main reason for this is related to the divergence towards infinity of the individual functions. For instance, in *Matlab* software, numerical values for $I_{n}\left(x\right)$ are only available for $x\leq700$; for higher values an infinity value is returned, and thus the ratio cannot be computed.

Interestingly, this ratio can be expressed in terms of Perron continued fraction [[70](#_ENREF_70)]:

$\frac{I_{n}\left(x\right)}{I_{n-1}\left(x\right)}=\frac{x}{2n+x-\frac{2x\left(n+1/2\right)}{2n+1+2x-\frac{2x\left(n+3/2\right)}{2n+2+2x-\frac{2x\left(n+5/2\right)}{2n+3+2x-\ddots}}}}$ (C-1)

A study about the convergence of this expansion [[70](#_ENREF_70)] revealed that it converges faster than other analogous representation based on Gauss continued fraction. For the purpose of this application, the summation in Eq. (C-1) is performed up to the final term $\frac{2x\left(n+5/2\right)}{2n+3+2x}$. Fig M in **S2 File** shows the accuracy of this approximation for different values of $n$ and for a wide range of values of $x$.

**D) dRL-SD+TV**

The dRL-SD method is based on the following iterative scheme [[37](#_ENREF_37)]:

$\mathbf{f}^{k+1}=\mathbf{f}^{k}\circ\mathbf{L}^{k}$, (D-1)

where

(D-2)

and $\mu$ is a parameter that depends on the standard deviation of the vector of measurements, $\mu=\max\left(0,1-4std\left(\mathbf{S}\right)\right)$. Notice that when the term $\mathbf{u}$ highlighted in (D-2) is equal to 1, the resulting damped version becomes equal to the original one described in (C-1).

The TV spatially-regularized extension to this method proposed in this work is based on the following modification:

$\mathbf{f}^{k+1}=\mathbf{f}^{k}\circ\mathbf{L}^{k}\circ\mathbf{R}^{k}$, (D-3)

where $\mathbf{R}^{k}$ is computed via Eq. (12) and the regularization parameter $\alpha_{TV}$ is computed following an approach similar to that used in RUMBA-SD+TV. Likewise, the noise variance is obtained by minimizing the negative Gaussian log-likelihood with respect to $\sigma^{2}$, which yields an iterative scheme analogous to Eq. (9).
