# Supplementary material for: Spherical Deconvolution of Multichannel Diffusion MRI Data with Non-Gaussian Noise Models and Spatial Regularization
Source: PLoS One. 2015 Oct 15;10(10):e0138910. doi: 10.1371/journal.pone.0138910 (PMC4607500; doi:10.1371/journal.pone.0138910)
Supplement: S2 File — (DOCX) [file pone.0138910.s002.docx]

**
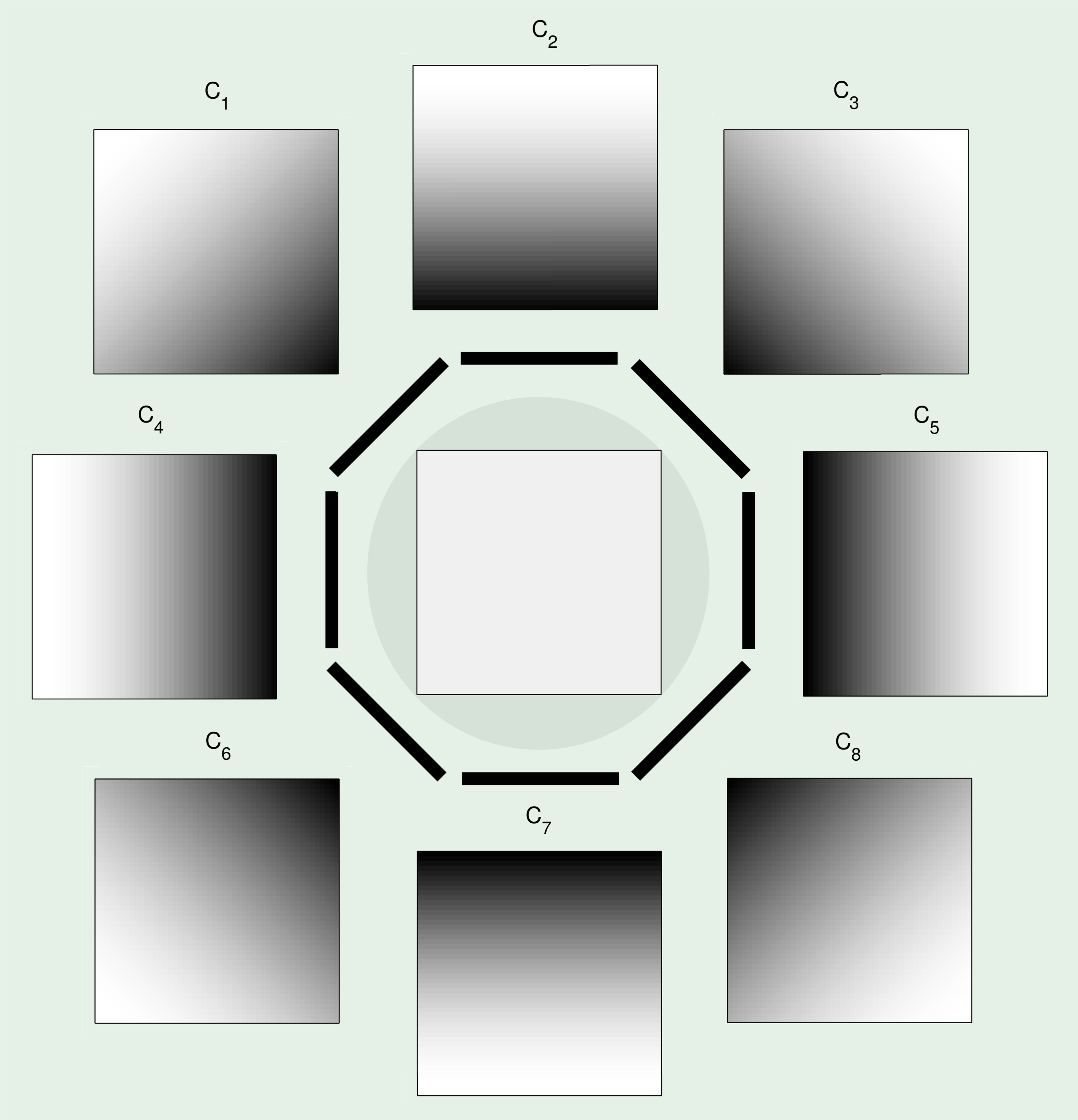
**

**Fig A**. Sensitivity maps simulating an eight-coil system. White colors denote higher values. The constant image in the center is the sum-of-squares of the individual sensitivity maps.


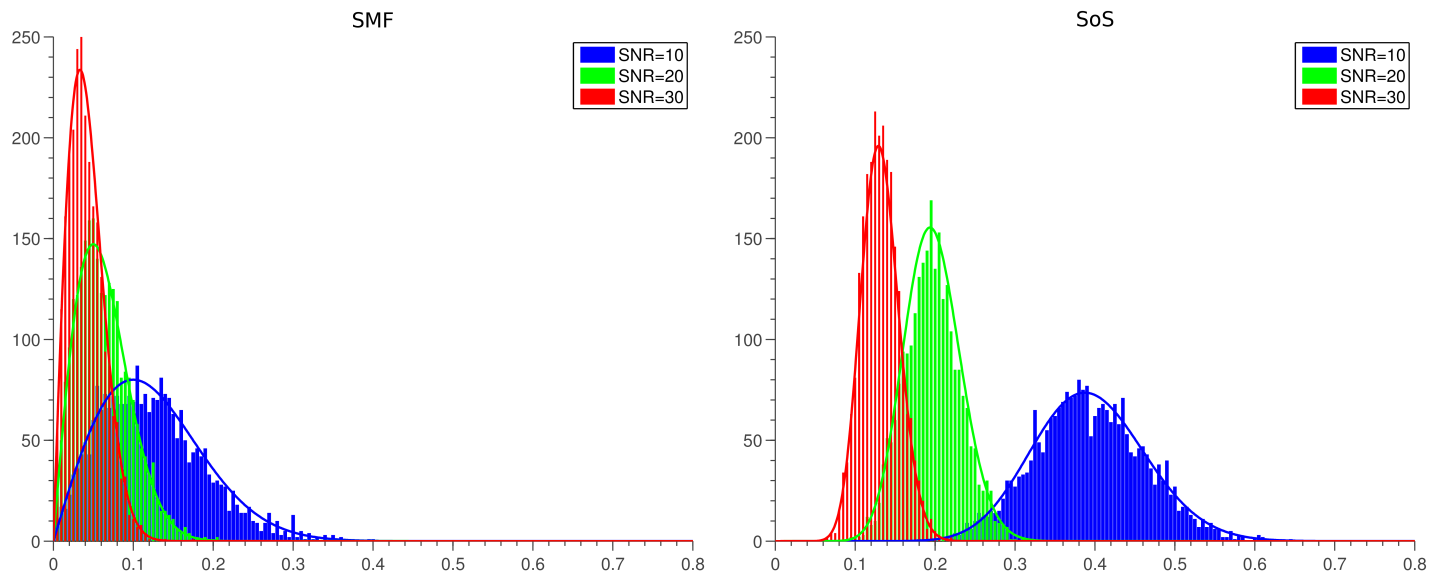


**Fig B**. Noise distribution profiles resulting from background regions outside the “HARDI Reconstruction Challenge 2013” phantom for the two reconstruction methods and SNR =10, 20 and 30. Rician and noncentral Chi distributions are obtained in SMF- and SoS-based data, respectively. The mean value of the distributions increases as long as the SNR decreases. This bias is higher in the SoS data.


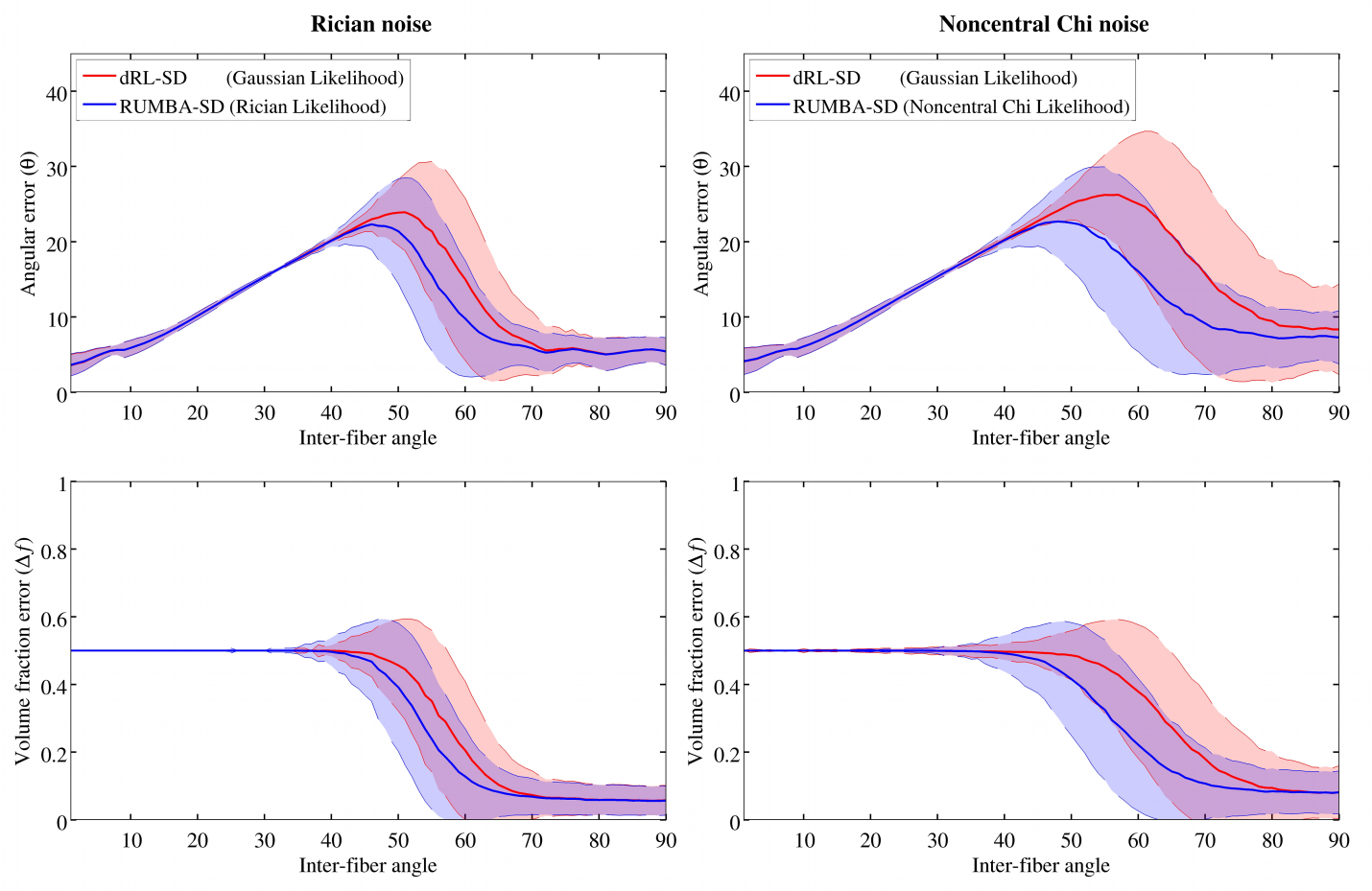


**Fig C.** Reconstruction accuracy levels for RUMBA-SD and dRL-SD using a dictionary based on estimated diffusivities. Reconstruction accuracy of RUMBA-SD (blue color) and dRL-SD (red color) is shown in terms of the angular error ($\text{θ}$) (see Eq. (17)) and the volume fraction error ($\Delta f$) (see Eq.(18)) as a function of the inter-fiber angle in the 90 synthetic phantoms. Continuous lines are mean values and semi-transparent coloured bands contain values within one standard deviation from the mean. Results are based on a dictionary created with empirical diffusivities from a diffusion tensor model applied on the data with a SNR = 15.


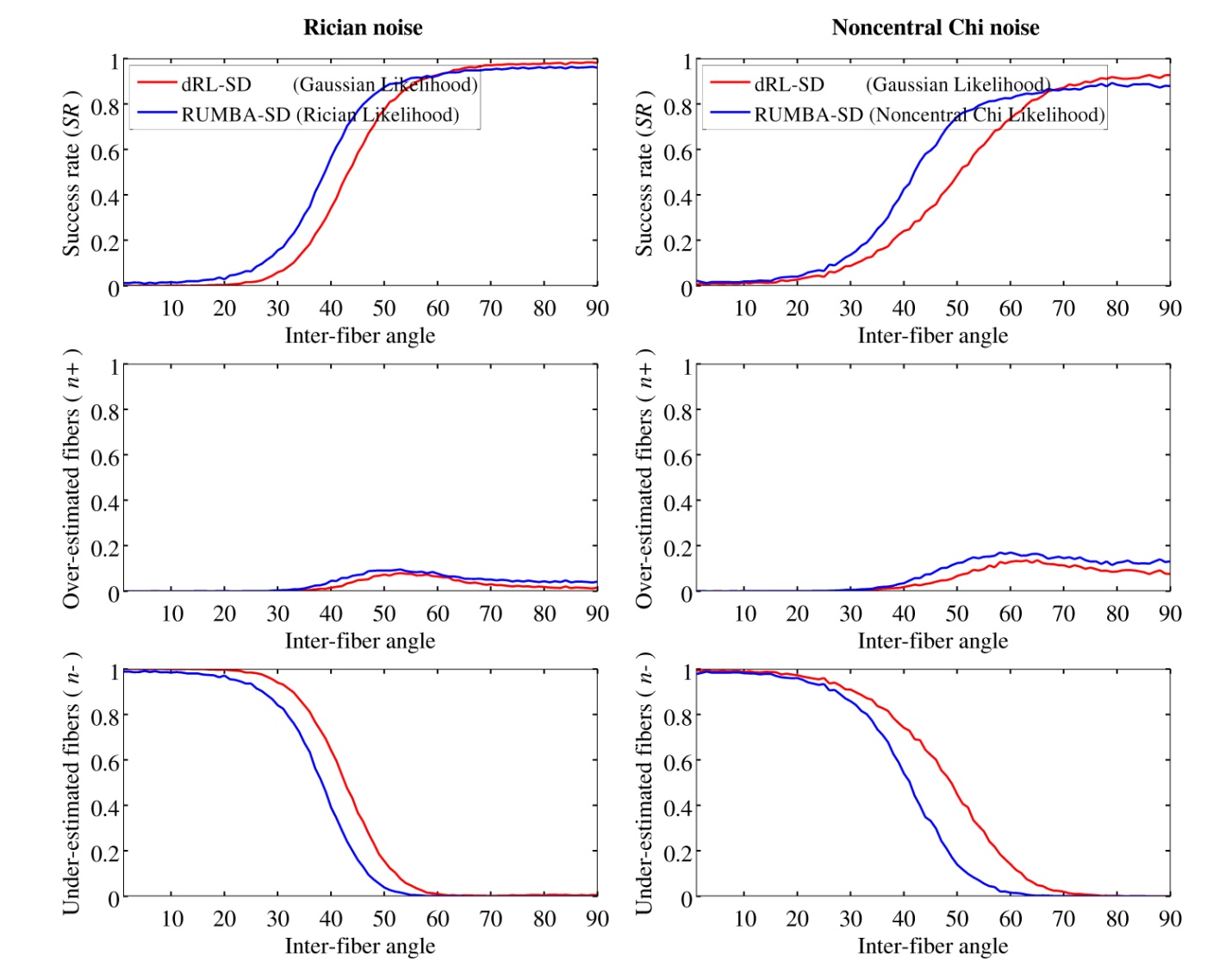


**Fig D**. Quantification of the reconstruction accuracy of RUMBA-SD (blue color) and dRL-SD (red color) in terms of the success rate (SR) and the mean number of over-estimated ($\text{n}^{+}$) and under-estimated ($\text{n}^{-}$) fiber populations, as a function of the inter-fiber angle in the 90 synthetic phantoms. The continuous lines in each plot represent the mean values for each method. This analysis refers to results using a dictionary created with the same diffusivities utilized to generate the data and a level of noise with a SNR = 15.

**
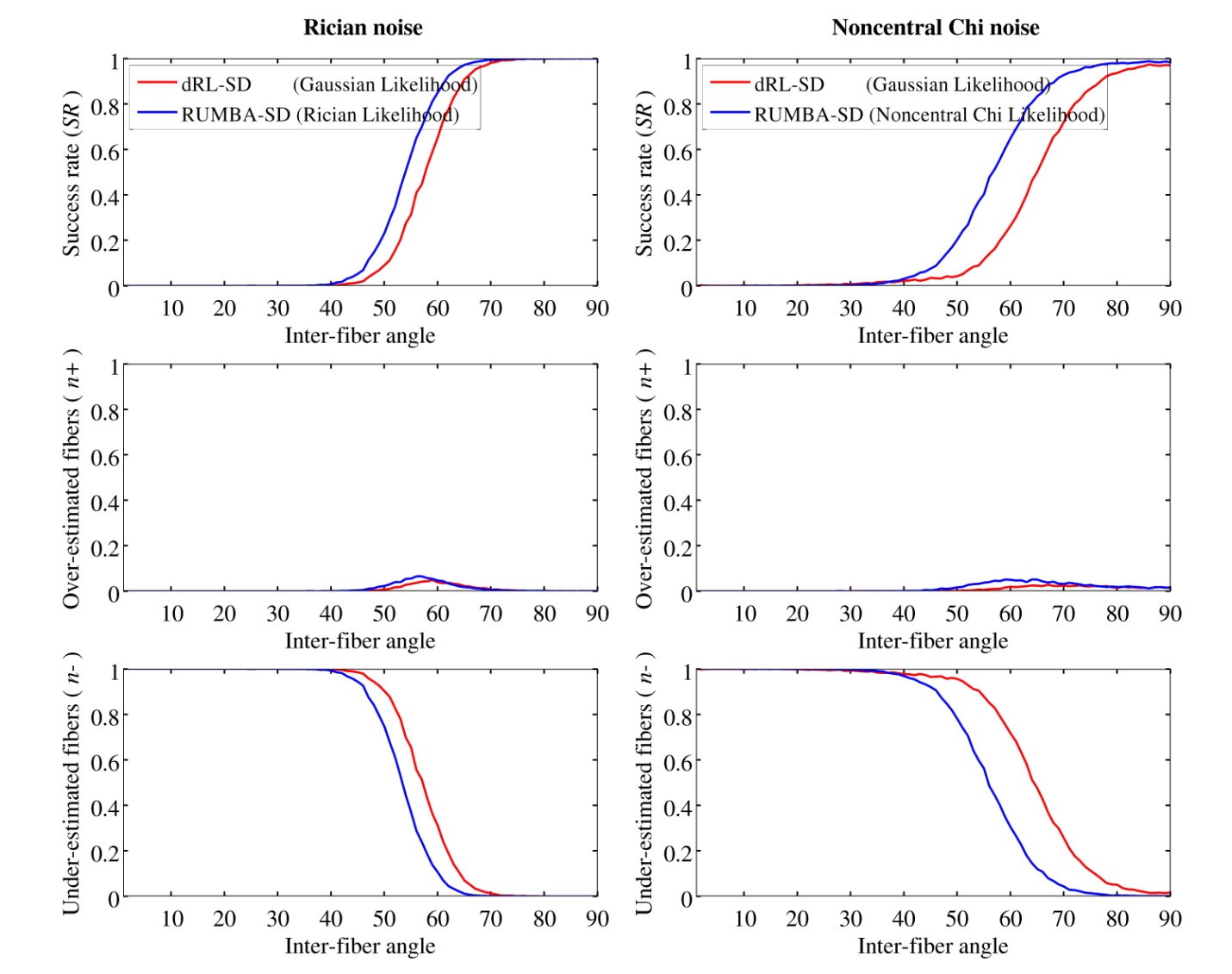
**

**Fig E**. Quantification of the reconstruction accuracy of RUMBA-SD (blue color) and dRL-SD (red color) in terms of the success rate (SR) and the mean number of over-estimated ($\text{n}^{+}$) and under-estimated ($\text{n}^{-}$) fiber populations, as a function of the inter-fiber angle in the 90 synthetic phantoms. The continuous lines in each plot represent the mean values for each method. This analysis refers to results using a dictionary created with empirical diffusivities estimated by means of the diffusion tensor model from the noisy data with a SNR = 15.

**
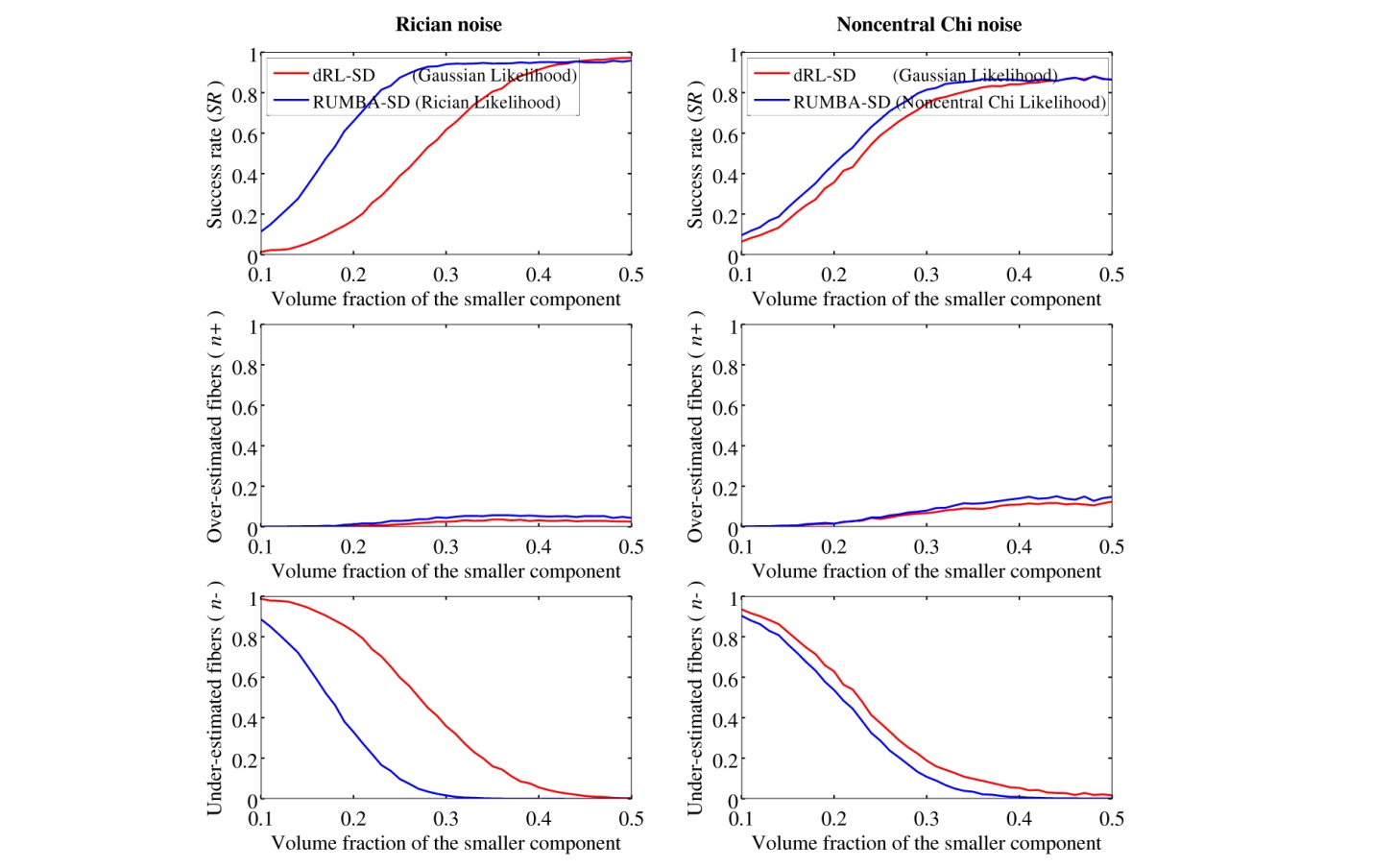
**

**Fig F**. Quantification of the reconstruction accuracy of RUMBA-SD (blue color) and dRL-SD (red color) in terms of the success rate (SR) and the mean number of over-estimated ($\text{n}^{+}$) and under-estimated ($\text{n}^{-}$) fiber populations, as a function of the volume fraction of the smaller fiber bundle in the 41 synthetic phantoms with inter-fiber angle equal to 70 degrees and different volume fractions. Results refer to the datasets with SNR=15 and dictionary created with the true diffusivities.

**
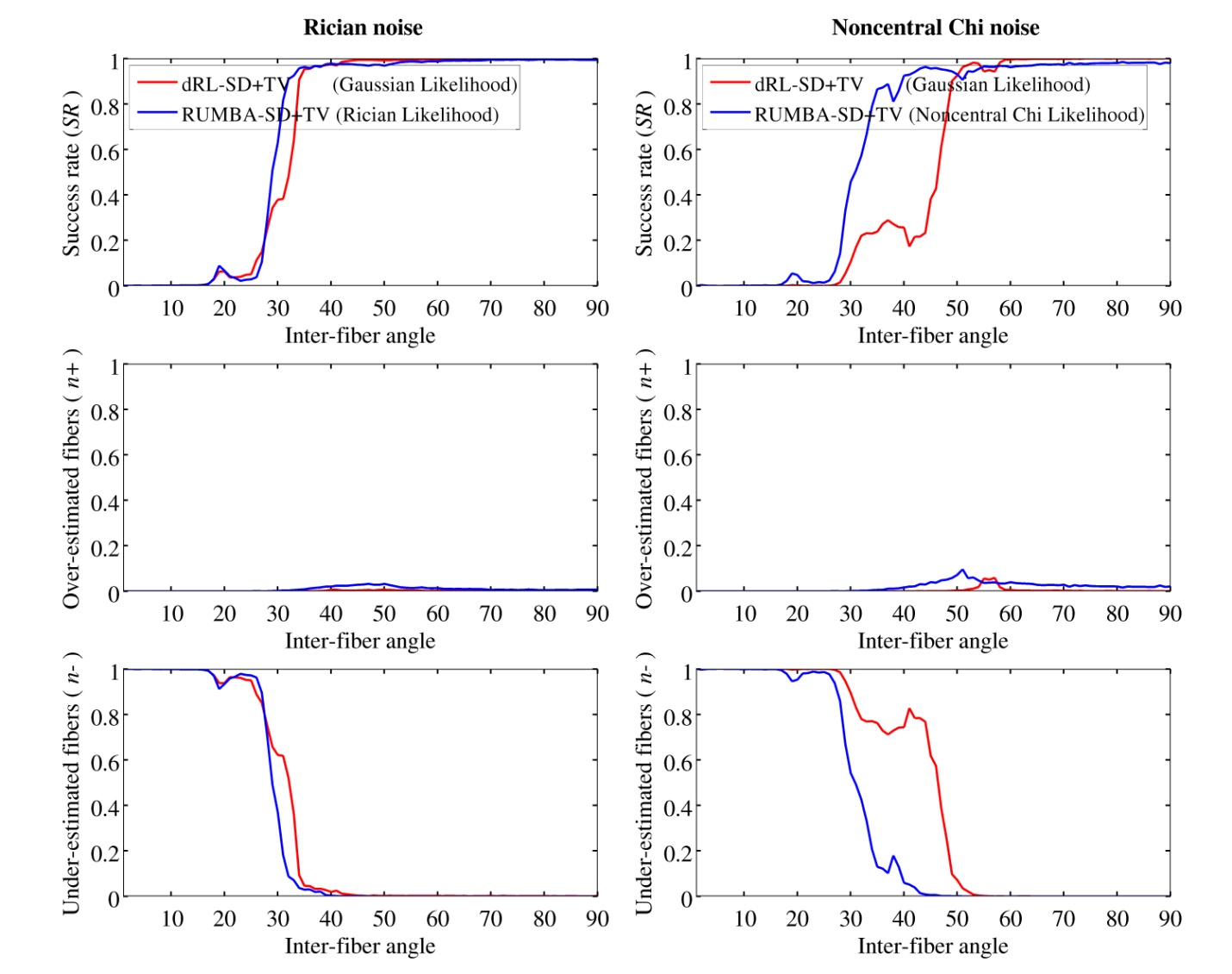
**

**Fig G**. Quantification of the reconstruction accuracy of RUMBA-SD+TV (blue color) and dRL-SD+TV (red color) in terms of the success rate (SR) and the mean number of over-estimated ($\text{n}^{+}$) and under-estimated ($\text{n}^{-}$) fiber populations, as a function of the inter-fiber angle in the 90 synthetic phantoms. The continuous lines in each plot represent the mean values for each method. This analysis refers to results using a dictionary created with the same diffusivities utilized to generate the data and a level of noise with a SNR = 15.

**
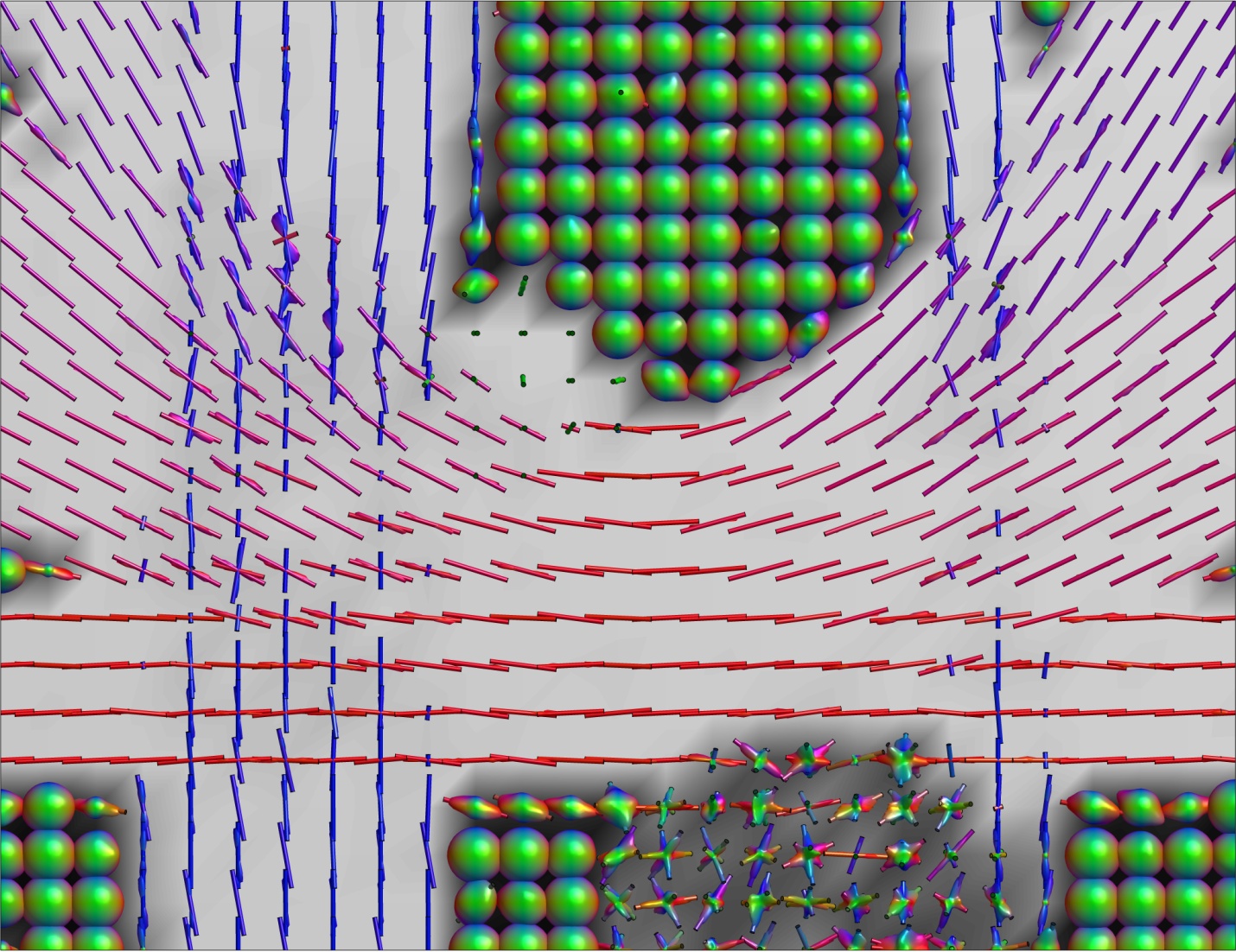
**

**Fig H**. Visualization of the fiber ODFs and their peaks (plotted as thin cylinders) reconstructed from the SMF-based data generated with SNR = 20 in a coronal slice of the “HARDI Reconstruction Challenge 2013” phantom. Depicted fiber ODF profiles correspond to the estimates from **dRL-SD** using 400 iterations.


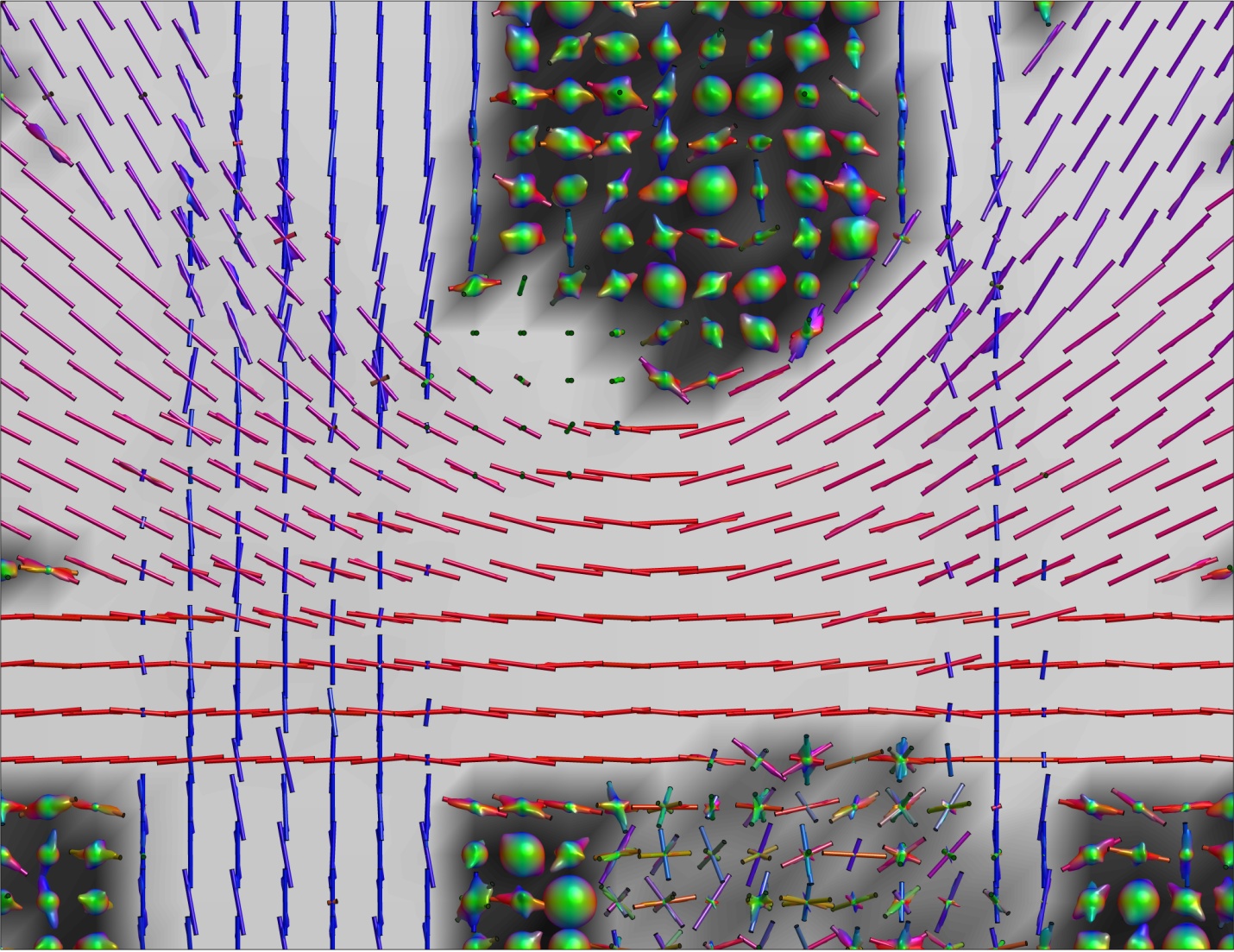


**Fig I**. Visualization of the fiber ODFs and their peaks (plotted as thin cylinders) reconstructed from the SMF-based data generated with SNR = 20 in a coronal slice of the “HARDI Reconstruction Challenge 2013” phantom. Depicted fiber ODF profiles correspond to the estimates from **RUMBA-SD** using 400 iterations.


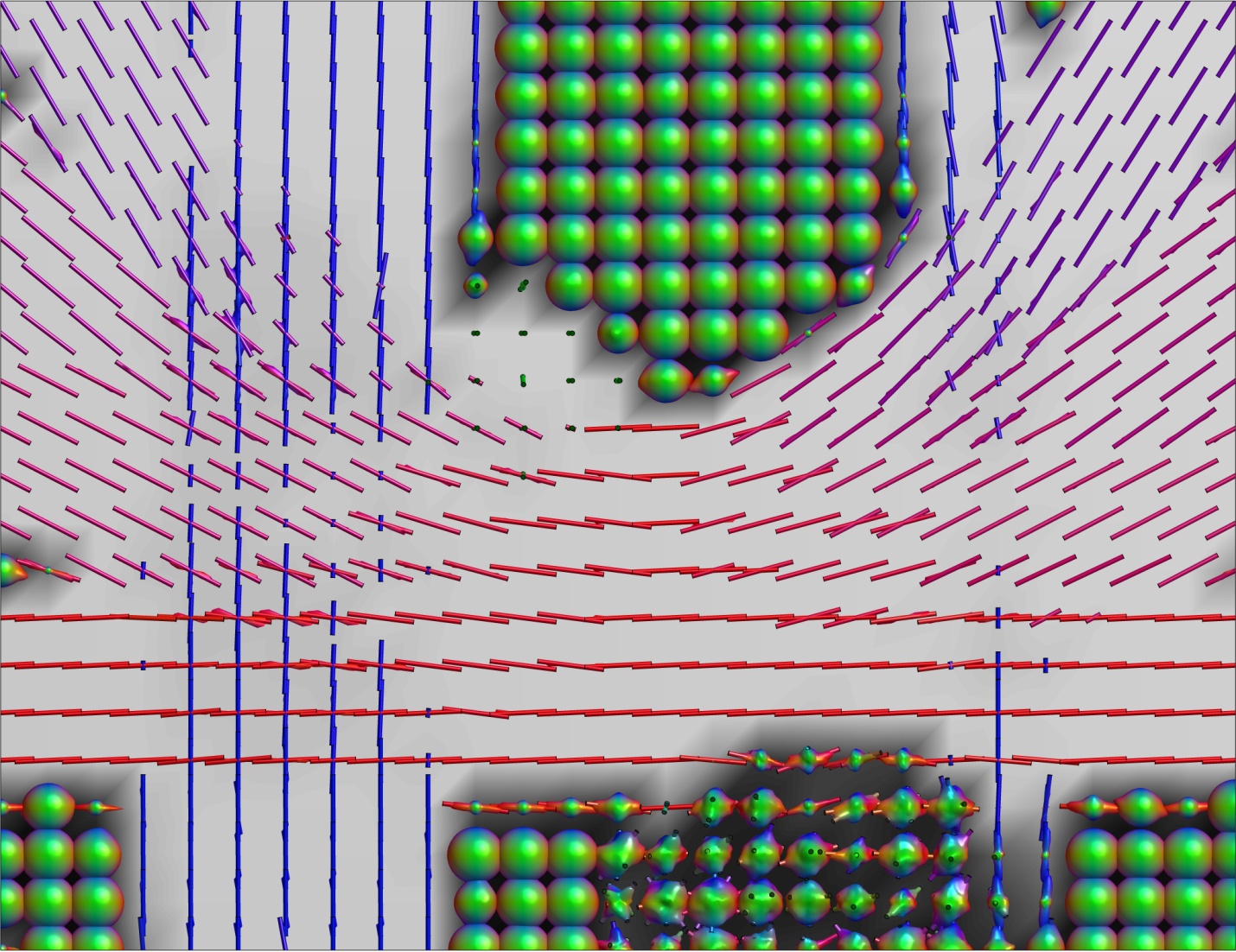


**Fig J**. Visualization of the fiber ODFs and their peaks (plotted as thin cylinders) reconstructed from the SMF-based data generated with SNR = 20 in a coronal slice of the “HARDI Reconstruction Challenge 2013” phantom. Depicted fiber ODF profiles correspond to the estimates from **dRL-SD+TV** using 400 iterations.


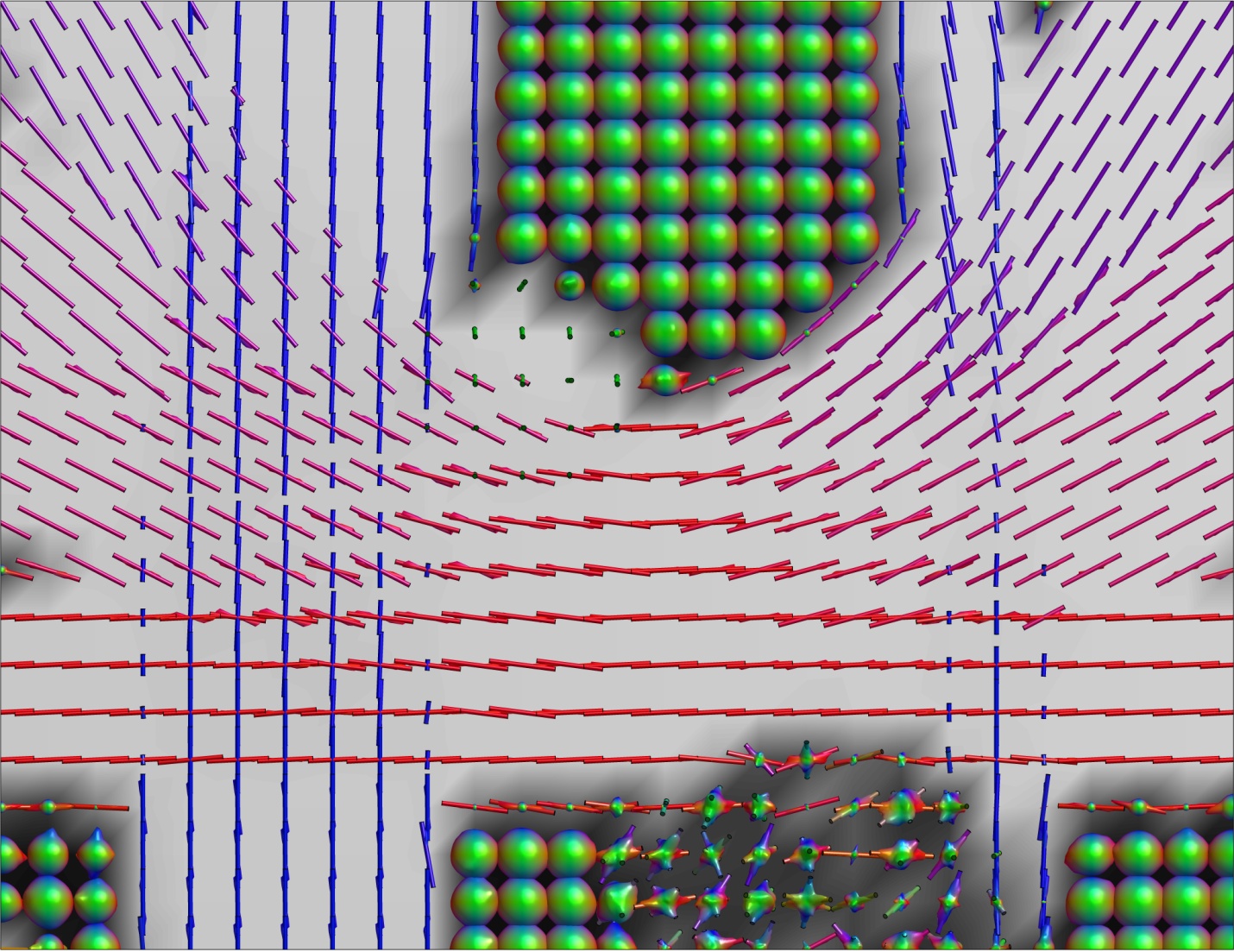


**Fig K**. Visualization of the fiber ODFs and their peaks (plotted as thin cylinders) reconstructed from the SMF-based data generated with SNR = 20 in a coronal slice of the “HARDI Reconstruction Challenge 2013” phantom. Depicted fiber ODF profiles correspond to the estimates from **RUMBA-SD+TV** using 400 iterations.

**
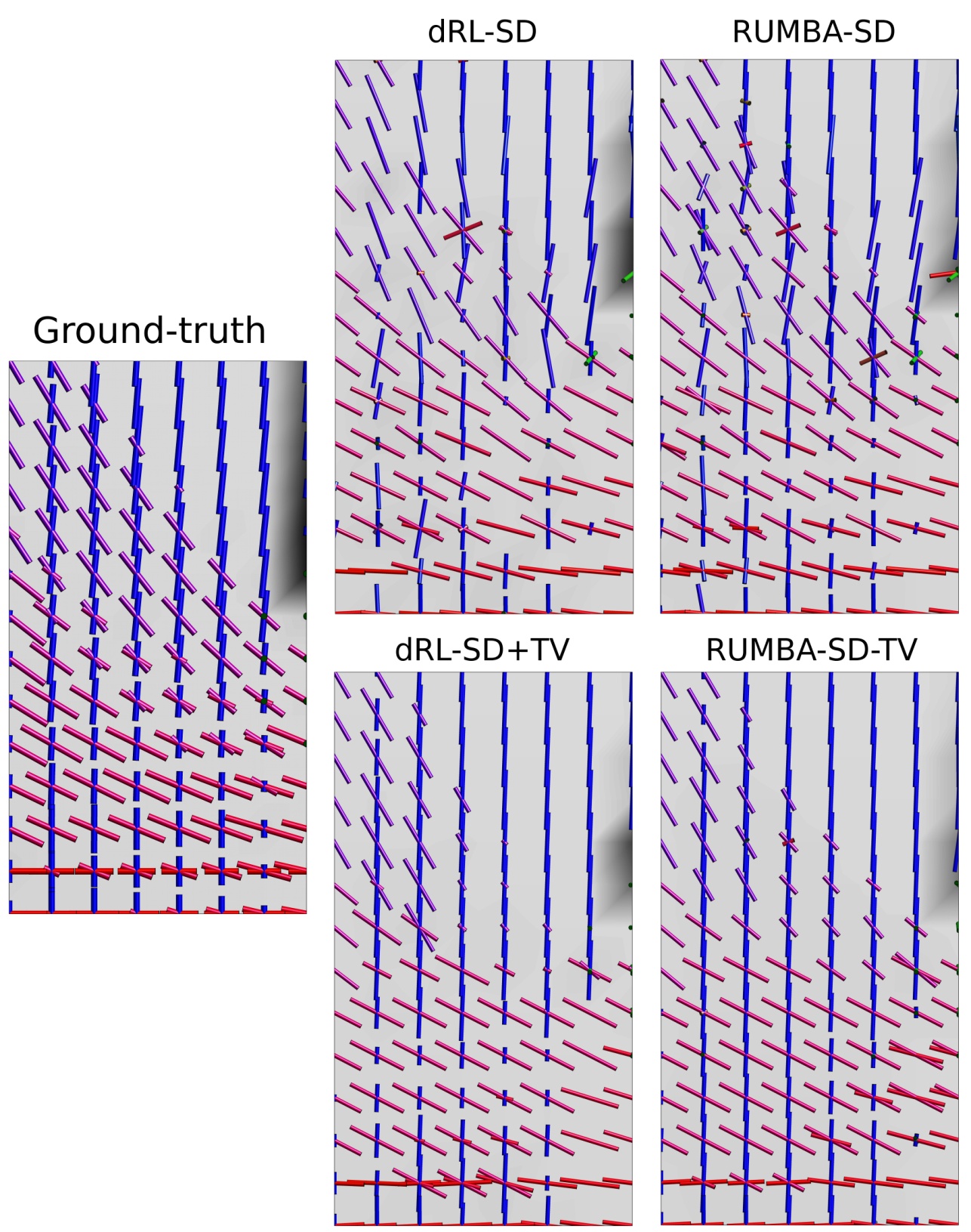
**

**Fig L**. Main peaks from the fiber ODFs estimated in the “HARDI Reconstruction Challenge 2013” phantom. Visualization of the main peaks extracted from the fiber ODFs reconstructed from the SMF-based data generated with SNR = 20 in a complex region of the “HARDI Reconstruction Challenge 2013” phantom. Results are based on reconstructions using 1000 iterations. Peaks are visualized as thin cylinders.


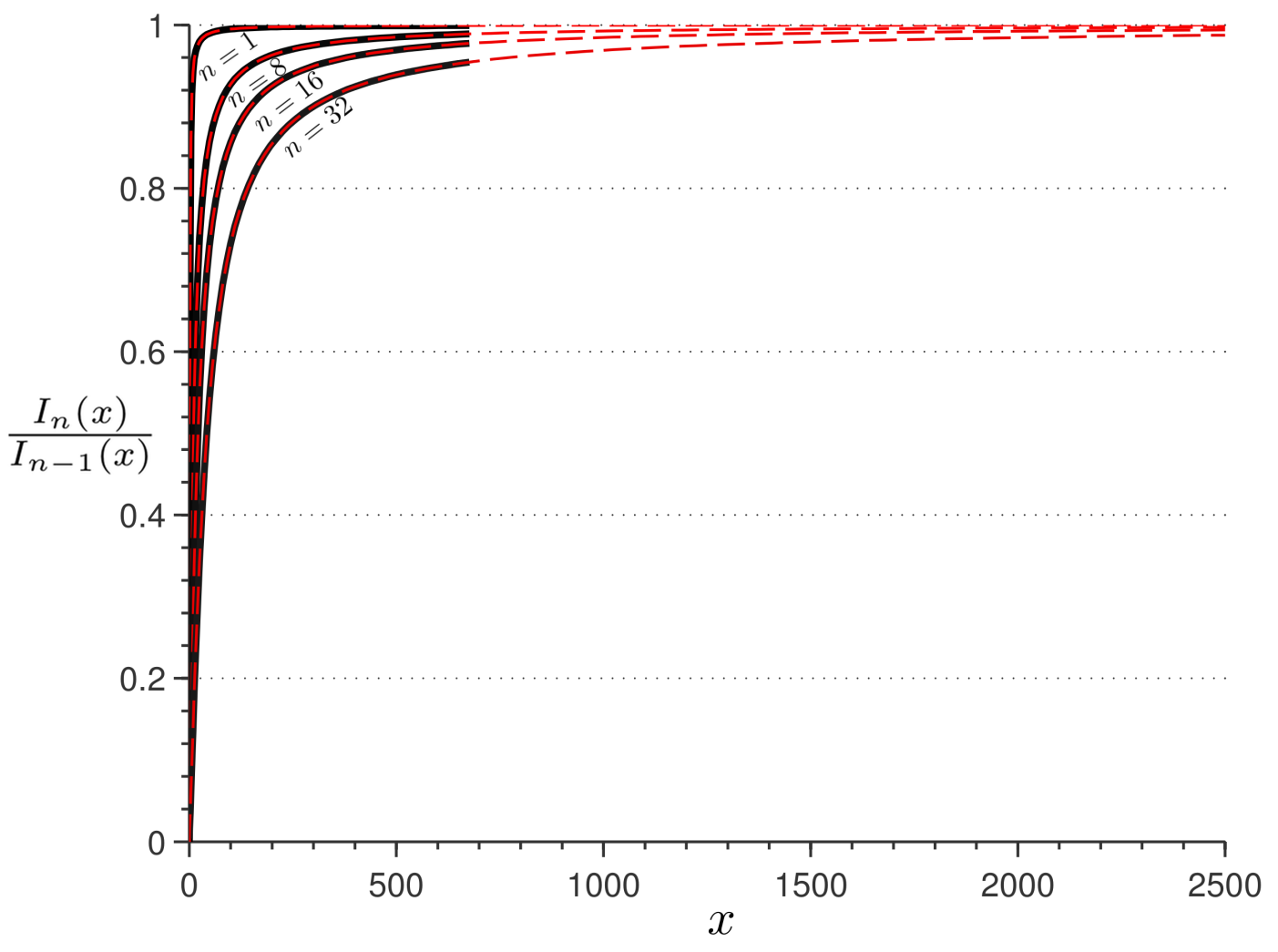


**Fig M**. Ratio of modified Bessel functions of first kind. Black continuous curves denote the exact values computed by means of the evaluation of the ratio of the individual Bessel functions. The fast divergence towards infinity of the individual functions does not allow evaluating this expression for the whole range of values. Red discontinuous curves denote the values computed by means of the Perron continued fraction approximation in Appendix C in S1 File, in the whole range of values.
